# Draft genome sequence of multidrug-resistant *Kurthia gibsonii* strain Hakim RU_BHWE isolated from sewage water in Bangladesh

**DOI:** 10.1128/mra.00546-24

**Published:** 2024-07-22

**Authors:** M. Romance, Md. Arif-Uz-Zaman Polash, Nusrat Zahan, Jafor Raihan, Md. Sumon Ali, Muhib Ullah khan, Subir Sarker, Md. Hakimul Haque

**Affiliations:** 1 Department of Veterinary and Animal Sciences, University of Rajshahi, Rajshahi, Bangladesh; 2 Biomedical Sciences and Molecular Biology, College of Public Health, Medical and Veterinary Sciences, James Cook University, Townsville, Australia; Department of Biology, Queens College, Queens, New York, USA

**Keywords:** whole genome, sewage water, multidrug-resistant, *Kurthia gibsonii*, Bangladesh

## Abstract

We have sequenced the genome of Kurthia *gibsonii* strain Hakim RU_BHWE, isolated from sewage water. The assembled genome consists of 2.891 Mb with 58.6883× coverage, presenting an average GC content of 36.60%. This genome includes 8 CRISPR arrays, 3 prophages, 3 antibiotic resistance genes, and 12 virulence factor genes.

## ANNOUNCEMENT

Since its discovery in 1883 by Hermann Kurth, *Kurthia* spp. has been known for its wide environmental distribution and its potential to cause opportunistic infections (1 – 5). Genome sequencing is crucial for understanding the survival, adaptation, and role of *Kurthia* spp. in antimicrobial resistance (6). Reports of multidrug-resistant *Kurthia* spp. in humans, animals, food, and the environment underscore the importance of ongoing surveillance through One Health approaches to understand its molecular epidemiology and implement effective public health strategies (1 – 3, 5, 7 – 9).

The research techniques and protocols for this study were approved by the Institute of Biological Science (IBSc) at the University of Rajshahi, Bangladesh, under Memo No. 56/321/IAMEBBC/IBSc. In September 2023, we collected samples of sewage water at the University of Rajshahi (24.3733°N, 88.6049°E), following standard procedures. The water samples were mixed thoroughly, transferred to sterile tubes, and transported to the laboratory. We then inoculated these samples on urinary tract infection agar (HiMedia, India) and incubated them aerobically at 37°C for 18–24 hours (10). *Kurthia gibsonii* was isolated by streaking the cultures on tryptic soy agar (HiMedia), followed by staining and biochemical tests (11). Antibiogram study of the isolates was performed using the disk diffusion method (12), following Clinical and Laboratory Standards Institute guidelines (13). The strain exhibited resistance to penicillin, amoxicillin, tetracycline, and doxycycline. We cultured the isolated strain in nutrient broth (HiMedia) overnight at 37°C and then extracted its genomic DNA using the Qiagen DNA Mini Kit (QIAGEN, Hilden, Germany). The genomic DNA was enzymatically fragmented using the NEBNext dsDNA Fragmentase Kit (NEB, Massachusetts, USA), and size selection was carried out with solid-phase reversible immobilization beads (14). A sequencing library was prepared using the Nextera DNA Flex Library Preparation Kit (Illumina, San Diego, CA, USA), and the library was sequenced with 2 × 150 paired-end reads on the Illumina NextSeq 2000 platform. Quality checks were performed using FastQC v.0.11.7 (15). Raw paired-end reads (*n* = 2,450,168) were trimmed using Trimmomatic v.0.39 (16), and genome assembly was conducted using Unicycler v.0.4.9 (17). The annotation of the genome was carried out using PGAP v.3.0 (18). The assembled genome was analyzed for antibiotic resistance genes (ARGs) using CARD v.3.2.4 with RGI v.6.0.2 (19) and ResFinder v.4.1 (20), mobile genetic elements (MGEs) using mobileOG-db (21), virulence factor genes using VFDB with VFanalyzer v.4.0 (22), pathogenicity index using PathogenFinder v.1.1 (23), sequence type using MLST v.2.0 (24), CRISPR arrays using CRISPRimmunity (25), prophages using PHASTER (26), and metabolic functional features using RAST v.2.0 (27). We used default parameters for all tools, unless noted otherwise.

**Fig 1:**
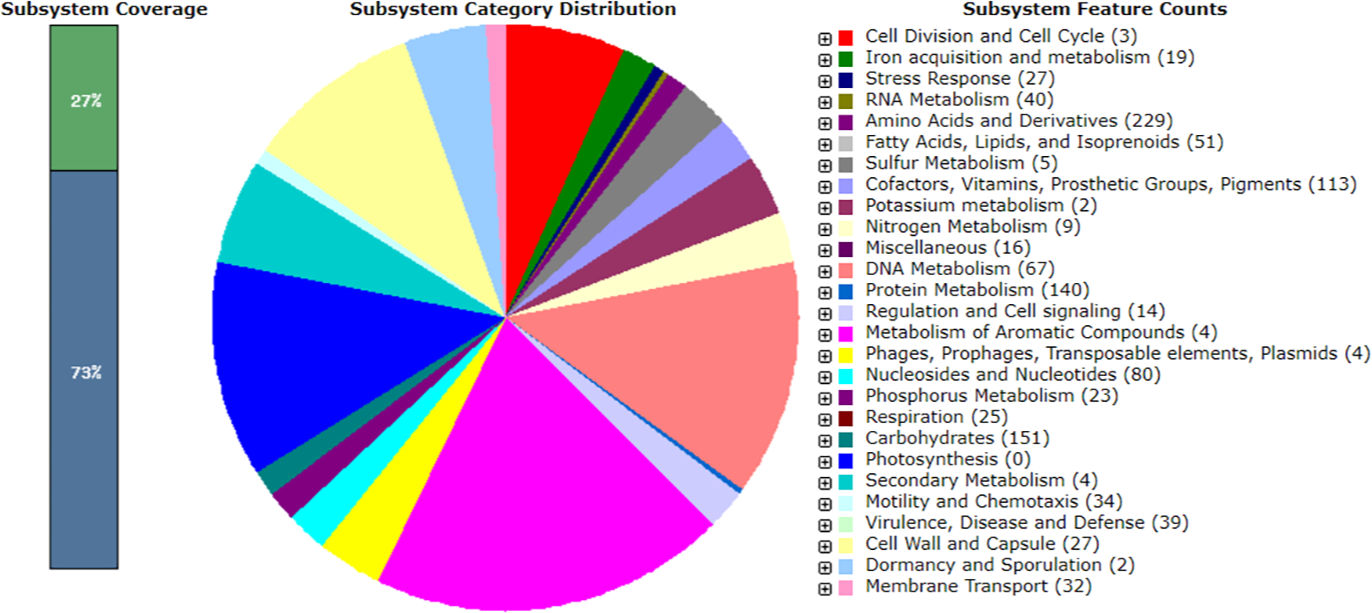
Metabolic functional features in the assembled genome of the *Kurthia gibsonii* strain Hakim RU_BHWE in SEED viewer. The 27% coverage indicates the completeness of functional roles within a specific subsystem across different genomes.

The traits of the draft genomes are documented in Table 1. Notably, 3 ARGs, 12 virulence genes, and 95 MGEs were predicted. MLST classified the genome as sequence type unknown. The genome exhibited eight CRISPR arrays with signature genes (*Cas14j*, *WYL*, *csa3*, *cas1*, *cas2*, *cas4*, *cas5*, *cas7, DEDDh*, and *cas8c*) and three prophages. RAST analysis uncovered 261 subsystems comprising 2,943 genes with 27% coverage (Fig. 1).

**TABLE 1:** Genomic traits of the *Kurthia* strain Hakim RU_BHWE

Elements	Values
Genome size	2,891,399 bp
Genome coverage	58.6883×
G + C content	36.60%
Number of contigs	104
Contig L50	10
Contig N50	101,097 bp
Total genes	2,920
Coding sequences	2,868
Coding genes	2,836
RNA genes	52
tRNA genes	44
rRNAs genes	3
ncRNAs genes	5
Pseudo genes	32
Genes assigned to SEED subsystems	2,943
Number of subsystems	261

## Data Availability

The study on Kurthia gibsonii strain Hakim RU_BHWE, conducted using the whole genome sequencing shotgun approach, was submitted to National Center for Biotechnology Information/GenBank, and the assembly was deposited under accession number JBCHWB000000000. The pertinent data, including the original readings, were stored with BioProject accession number PRJNA1102855, BioSample accession number SAMN41030973, and Sequence Read Archive accession number SRR28762083. The specific version mentioned in this document is labeled as JBCHWB000000000.1.
